# Is participation in high-status culture a signal of trustworthiness?

**DOI:** 10.1371/journal.pone.0232674

**Published:** 2020-05-05

**Authors:** Amelie Aidenberger, Heiko Rauhut, Jörg Rössel

**Affiliations:** 1 Institute of Sociology, University of Zurich, Zurich, Switzerland; 2 School of Sociology, University College Dublin, Dublin, Ireland; Middlesex University, UNITED KINGDOM

## Abstract

Trust is essential for social interactions, cooperation and social order. Research has shown that social status and common group memberships are important determinants of receiving and reciprocating trust. However, social status and group membership can coincide or diverge–with potentially different effects. Our study contributes to the existing literature on the role of status and group membership by testing two separate trust-generating mechanisms against each other. We examine if individuals tend to place trust in high-status groups (irrespective of their own group membership) or, rather, if they tend to trust others with whom they share a common group membership. We assume that status group membership is signalled by cultural (musical) taste. This operationalization follows the theoretical reasoning of Bourdieu who argues that it is, above all, musical taste that classifies persons of different status. By demonstrating their “legitimate” cultural taste, upper-class members distinguish themselves from the middle and lower classes and signal their social status, thereby creating awe, respect and an air of trustworthiness. We report evidence from online experiments with incentivized trust games, which enable us to separate the two trust-generating mechanisms. We find no evidence that persons with “legitimate” tastes are generally trusted more. Instead, our results clearly demonstrate ingroup favouritism towards persons with a similar taste. Participants place more trust in members of their own group and expect them to be more trustworthy. In other words: members of taste-based groups trust each other more than members of different-taste-based groups. Interestingly, this group-based trust is not always justified inasmuch as received trust is not necessarily reciprocated more strongly by own group members. This suggests that ingroup favouritism is, at least in part, driven by false beliefs.

## Introduction

Placing trust in trustworthy individuals is key for establishing collective goods, cooperation and collaboration and has far-reaching consequences for the order, development and prosperity of societies [1,2]. Since different individuals in different situations vary considerably in their level of trustworthiness and cooperativeness [3,4], choices of whom to trust have to be made on many occasions in every individual’s daily life and can, in the case of misplaced trust, result in substantial losses. Making well-informed decisions may be easy when we have previous experiences with our interaction partners. In contemporary societies, however, placing trust in the right people has become increasingly demanding. With progressing economic and political globalisation, growing mobility and increasing numbers of connections with weak ties, more and more interactions take place between strangers [5–8]. Thus, in many situations, individuals do not have sufficient information on whether their interaction partners are trustworthy or not.

Decisions of whom to trust can be based on a wide range of cues and characteristics, such as personal traits of the trustee–for instance, his or her voice–, the nature of the relationship between trustor and trustee or situational aspects, such as the mood of the involved individuals [9,10]. Sociologically more relevant, however, are social characteristics, such as social status and group membership of the trustor and the trustee [11]. In the present study, we focus on this aspect.

We consider two ways in which membership of social groups may affect trust and trustworthiness: We investigate if individuals trust others with high social status or, rather, others with whom they share a mutual social group. Following extant work in sociology [12,13], we assume that status group membership is signalled via cultural tastes–in our case, musical preferences. Thus, we do not focus on very specific forms of social status (such as the status that comes with being a celebrity or politician). Instead, we measure status in general terms by employing music preferences as a proxy for perceived social status in terms of education, income and occupation, which are conventionally used in the social sciences to measure social status for all members of contemporary societies [14]. High-status culture is often operationalized via a preference for classical music. Thus, we study one group of classical music lovers and one of folk music lovers. This allows an empirical comparison of two competing mechanisms: are people with high-status tastes trusted more (irrespective of one’s own status) or do people trust others with the same group membership more (independently of whether they have high or low social status)? In other words, we test a signalling mechanism (cultural taste as a signal of status group membership) against ingroup favouritism. Both mechanisms may work together in real social life–however, our experimental design enables us to disentangle them empirically.

While only few studies have shed light on the relevance of group status for trusting behaviour [15,16], a wide range of empirical investigations has examined the role of ingroup favouritism [17–19]. However, no attempt has been made thus far to consider both perspectives simultaneously and to systematically test both mechanisms against each other. The aim of the present study is to contribute to the existing literature by closing this gap. We reveal if and how belonging to a social group affects an individual’s perceived and actual trustworthiness. Specifically, we shed light on whether one particular social group–signalling high social status–appears and acts more trustworthy than another–signalling lower status–or, rather, if trust operates on the basis of shared group memberships.

The remainder of the article is structured as follows: we first outline our theoretical considerations regarding status as a basis of trust as well as concerning cultural preferences as a signal of social status and ingroup favouritism. From these considerations, we derive our research hypotheses about musical preferences as a signal of social status, social status as a signal of trustworthiness and ingroup favouritism. Subsequently, we illustrate the experimental design and our data collection. We then present the results of our trust game experiment, discuss our findings and draw conclusions for the literature on trust, cultural sociology, and social inequality.

## Theoretical background

We juxtapose two lines of theoretical reasoning: trust may either be driven by the signalling of social status or by shared group memberships. The former mechanism implies that individuals tend to trust members of one *particularly trustworthy* group–irrespectively of their own group affiliation. The latter suggests that persons place trust in others with whom they share a mutual social category. In the following sections, we present both theoretical arguments and the respective empirical literature.

### Signalling of social status as a basis for trustworthiness

One way in which group-based trust could operate is that the majority of people trust members of one specific group above average. From this perspective, decisions to trust another person are shaped by the trustee’s group, being considered as generally trustworthy or untrustworthy. This implies that placing trust is independent of the trustor’s own group membership. Empirical research has shown that individuals tend to hold positive views of high-status groups [20]. Thus, we suggest that status is a basis of trustworthiness.

High social status correlates with benevolent and cooperative behaviours and, thus, trustworthiness. There are a number of arguments for this relationship. From a rational-economic perspective, high-status individuals are better-endowed with resources. This implies that they have the financial and cognitive means to act in favour of others, experience less pressure to maximize their own resources and have fewer incentives to exploit others [21–23]. This reasoning is corroborated by strain theory [24]: those who are well off are able to conform with (cooperation) norms while the disadvantaged only have the option to deviate in order to achieve their goals. From this point of view, high-status individuals comply more strongly with moral standards, such as reciprocating trust, than low-status individuals. A similar argument can be made based on the social norm of *noblesse oblige* [25]. Here, the association between high social status and trustworthiness is generated by the moral obligation of “those of higher rank to be honorable and generous in their dealings with those of lower rank” (25, p. 320). Thus, high-status individuals experience stronger societal pressures to act in a socially responsible manner, which includes behaving fairly and cooperatively and reciprocating trust [22,26,27]. Taken together, these theoretical considerations suggest that high social status correlates with high levels of trustworthiness.

There are a number of studies indicating that social status and trust in fact correlate. However, only a handful were *explicitly* designed to examine the impact of group status on trustfulness and trustworthiness. A pioneering study of status and trust is a field experiment by Falk and Zehnder [15]. They conducted a trust game experiment with participants from different residential districts in Zurich, either characterised by high or low socioeconomic status. Participants could decide how much of their endowment in the game they would like to send to the person they were matched with–conditional upon the district the receiver lived in. The results showed that subjects tended to invest more money–and, thus, to place more trust–into residents of high-status areas. In addition, findings showed that persons from high-status districts were also more willing to reciprocate trust by repaying their investor, which implies that residents of wealthy districts were not only perceived as more trustworthy, but indeed proved to be more reliable interaction partners. Somewhat similar results were produced in a study by Cox and colleagues [28]. They ran public-goods games in rural India, bringing together participants from high- and low-caste backgrounds. In those conditions where participants were informed about others’ caste background, cooperation was highest among groups made up only of high-status participants and lowest among groups consisting solely of low-status participants; results for mixed groups were in-between. These findings, in general, indicate higher trustworthiness of higher-status groups. Remarkably, these studies–as our own research endeavour–are rare examples of relevant previous research conducted with a non-student sample, increasing the generalisability of results. Further empirical inquiries related to our research include those of Trifiletti and Capozza [29], who performed a trust game with participants from low-status Southern Italy and high-status Northern Italy and observed that low-status group members trusted high-status group members more. However, high-status participants proved to be the more tolerant players: unlike low-status subjects, they did not differentiate by status, but rather trusted low- and high-status group members similarly. Employing the same paradigm, Lei and Vesely [16] induced artificial income differences in a laboratory experiment. Thus, this research differs from Falk and Zehnder [15], Cox and colleagues [28] and Trifiletti and Capozza [29]–as well as from the present study–in one central aspect: here, the status differences were no real-life disparities, but fabricated by the researchers for the duration of the experiment. Findings showed that subjects trusted ‘rich’ (high-status) participants more than ‘poor’ (low-status) ones. This status-effect was observed for both low- and high-status subjects and persisted even when initial income inequalities were removed. Qi, Li and Du [30] also conducted a trust game experiment with artificial income differences. In a first step, the authors let participants rate the trustworthiness of persons shown in photographs and provided (fictitious) information about the displayed person’s income. Here, the authors found that more affluent characters were rated more trustworthy than those with lower incomes. In a second step, they examined if these judgements would also lead to differential investments in a trust game and concluded that, indeed, high-income individuals were not only rated as more trustworthy, but also received more money in the game. As a last example from the realm of trust games, Berger [31] studied whether individuals displaying a high willingness to pay for eco-friendly products were trusted more than those preferring cheap but non-green products. Results showed that persons who paid a ‘green premium’ received more trust and also reciprocated trust more strongly.

Also scientists employing research methods other than trust game experiments have shown a relationship between social status and perceived trustworthiness. For instance, Keijzer and Corten [32] conducted a vignette experiment designed to simulate peer-to-peer market platforms where participants had to make trusting decisions based on a potential seller’s characteristics, including socioeconomic status (education and occupational prestige). Findings showed that higher status led to higher perceived trustworthiness; in addition, they revealed that high-status individuals were rewarded more for positive reviews for past selling exchanges than low-status actors. Even more general evidence is produced by survey studies, which show that persons of higher status, usually measured by education, are more trusting (see [33] for an overview). However, these studies do not analyse trustworthiness of individuals.

Nonetheless, it has to be noted that there is also research indicating that individuals with high social status tend to be less cooperative interaction partners. For example, Piff and colleagues [34] conducted four separate studies to investigate how participants’ social status (measured through self-reported subjective social status and/or based on their income) correlated with their prosociality. Results revealed that lower social status is associated with more prosocial behaviour: compared to high-status subjects, low-status participants were more generous (in a dictator game), declared greater support for charity (when asked which percentage of their salary people should spend on charitable donations), expressed more trust in strangers (via investments in a trust game) and were more likely to help another person in distress (by voluntarily taking on time-consuming tasks). Another strand of research has investigated the corrupting effects of power on individuals’ prosociality. In her review of the relevant empirical literature, Fiske [35] concludes that persons in power–who usually have high social status–tend to be egocentric, fail to take the needs of others (particularly those of lower status) into account, discriminate against their subordinates and exploit others. This is, for instance, also illustrated by the findings of Bendahan and colleagues [36], who conducted an experiment in which they employed variations of the dictator game to examine if more power would lead to higher levels of antisocial behaviour. Subjects were randomly divided into powerful ‘leaders’ and powerless ‘followers’. The researchers then bestowed power on the leaders by assigning them followers (either one or three) and three or four choices of how to divide money between themselves and their followers. The three choices were *altruistic*, *fair* and *antisocial* distributions of how to divide the money. The four choices included in addition a *very antisocial* option. Results showed that with increasing power (more followers, more choices), subjects’ tendencies to exploit their followers by choosing the antisocial (and very antisocial, if available) option grew significantly. This experimental research coincides with older case study research on urban working-class communities, emphasizing their reliance on solidarity and trust within social networks of family, kins and neighbours [37,38]. These results emerged in contrast to the middle-class perceptions of such neighbourhoods as disorganized slums and indicated the high degree of trust available and necessary to conduct everyday live in such poor communities.

To summarise, there is empirical evidence for both a positive and a negative effect of social status on trust and cooperativeness. The present research aims at contributing to this ongoing debate.

In light of our theoretical considerations and the empirical evidence showing that persons of high status are perceived as more trustworthy and also reciprocate trust more often, we feel confident to formulate the hypothesis that social status positively correlates with the likelihood of being trusted and repaying that trust. We test this empirically in our experimental research.

However, social status is not directly observable in many situations. For instance, persons in contemporary societies usually do not follow a formal dress code, but dress casually in their everyday lives [39]. Yet, there are a range of indicators which reveal and signal social status and, thus,–possibly–one’s trustworthiness and cooperativeness [40]. These indicators can, in the terminology of signalling theory, either be seen as inadvertent *signs* or deliberate *signals*. While signs are “anything in the environment that is perceptible and by being perceived happens to modify our beliefs about […] someone” (36, p. 170), signals are “observable features of an agent which are intentionally displayed for the purpose of raising the probability the receiver assigns to a certain state of affairs” (ibid.). Here, we argue that participation in high-brow culture may either be a sign (merely correlating with behaviour) or a signal (i.e., an intentional demonstration) of high social status and, thus, of trustworthiness. By the term high-brow culture, we refer mainly to the classical forms of art like classical and chamber music, visual arts, opera, theatre and ballets in western societies. It is not assumed that these are in any way intrinsically aesthetically superior to popular and folk culture. The term high-brow culture refers to the fact that these forms of art are still consumed by highly educated groups with high social status and are seen as a signal of status [41]. This is clearly the case in Switzerland, where our experiment was conducted. Survey evidence shows that a preference for classical music is seen as one of the strongest differences between higher social classes, who prefer classical music, and lower social classes, who do not [42]. Furthermore, Swiss cultural discourse in leading quality newspapers still focuses more on high-brow culture. Moreover, cultural policy mainly supports classical culture like classical orchestras, theatres, museums and operas, on which more public funds are spent than on all other leisure and sports activities combined [43,44].

There are theoretical and empirical arguments that consumption of high-brow culture is a *sign* of social status: following Bourdieu [13], cultural preferences are a central component of an individual’s habitus, which is shaped by social class membership. Bourdieu [13] demonstrates that the dominant groups in society can be identified by their cultural practices, e.g. a preference for classical music, and their ability to aestheticize all forms of art. By consuming culture in accordance with this aesthetic attitude, upper-middle class and upper-class individuals are able to distinguish themselves from members of the lower classes, to show their adherence to and familiarity with “legitimate” (classical) culture and to evoke admiration and reverence from members of the lower classes. This “legitimate” culture is also supported by its institutionalization in cultural policy, educational systems and public discourse. Bourdieu uses this term despite its normative connotation to critically identify the processes that transform the cultural preferences of the higher status groups into a dominant culture that creates widespread awe in society. In his empirical studies on the aesthetics and lifestyles of different social classes, Bourdieu shows that it is, above all, musical taste that classifies persons: “…nothing more clearly affirms one’s class, nothing more infallibly classifies, than tastes in music” (13, p. 18). In Bourdieu’s approach, the dominant cultural order is internalised by all members of society and is habitually enacted. This implies that an aesthetic preference for high-brow culture is not an intentionally chosen signal of high social status, but an inadvertent sign thereof. Note that there is an ongoing discussion if classical highbrow culture is still a sign of high social status in contemporary societies [45]. We do not want to contribute to this discussion and with regard to this study we stick to Bourdieu’s [13] original formation and take classical music as a strong sign of high social status.

On the other hand, there are arguments from signalling theory that displays of musical tastes can be regarded as intentional *signals* of social status. To illustrate this, we briefly outline what we mean by signalling. We follow Gambetta [46] by specifying two criteria for a signalling sequence. “(i) There is some action the receiver can do which benefits a signaller, whether or not he has the quality k […] (ii) this action benefits the receiver if and only if the signaller truly has k, and otherwise hurts her”. With respect to status and trust, this means: a signaller (the trustee) will benefit from being trusted, whether or not she actually is trustworthy. However, the receiver (the trustor) benefits from trusting the signaller if–and only if–he or she truly has this quality. Situations of this type may lead to a sorting equilibrium if an honest signaller has lower costs for signalling a certain unobservable quality compared to a dishonest signaller. On the premise that consuming high-brow culture is associated with lower costs for individuals with high status–for instance, because of their socialisation, education, available time and ability to appreciate such arts–, it appears plausible that high-brow culture consumption is an effective signal for status (and, thus, trustworthiness) *only* if the signaller truly possesses this trait. Thus, a sorting equilibrium, in our case, is based on the existence of true beliefs on the relationship between cultural tastes, social status and trustworthiness. We will analyse the existence of such true beliefs as part of our experimental study.

Whether an inadvertent sign or a deliberate signal, an association between classical music consumption and (perceived) socioeconomic status has been observed in numerous empirical investigations [42,43,47–50]. Thus, the preference for classical music demonstrates membership of the higher-status groups and can be assumed to create awe, respect and trust in all members of society. But not only the dominating classes, but also the lower-status groups are characterised by their cultural tastes. While the former lean towards high-brow arts, the latter tend to exhibit more popular preferences. The lower-status groups thus favour art forms designed to offer simple and lightweight entertainment [13,51]. In the realm of music, this encompasses genres featuring casual, easy-going rhythms, instruments and lyrics, such as traditional folk music (‘Volksmusik’). An empirical link between a fondness of folk music and lower socioeconomic status, particularly lower educational levels, has been shown in a number of studies [48,52–55]. Therefore, we employ a preference for folk music as a sign of low-class membership.

In addition to relying on the literature on the relationship of individuals’ music tastes and their (perceived) social status, we complement findings of previous research with data from our own sample. We analysed whether lovers of folk and classical music are perceived differently in terms of their social status by the subjects in our study. We can show that lovers of classical music are indeed seen as being of higher status by both folk fans and classic enthusiasts (detailed results are provided in the results section).

In sum, we deduce our first two propositions from the reasoning outlined above:

#### Status perception hypothesis

*Consumers of high-brow culture are attributed higher social status by others, irrespectively of the observer’s own cultural preferences*.

#### Status hypothesis

*Consumers of high-brow culture (i) are perceived as more trustworthy and (ii) behave more trustworthy compared to consumers of low-brow culture*.

### Ingroup favouritism and trust

In contrast to the reasoning outlined above, one can also argue that group-based trust operates on grounds of *common* group memberships and that individuals are more likely to place trust in others with whom they share a group identity. This mechanism is known as *ingroup favouritism* or *ingroup bias* [56–59]. It describes individuals’ tendency to treat others with whom they share group identities (members of their *ingroup*) differently from people they have no group membership in common with. They tend to hold more positive views of ingroup members [56,60,61] and treat them more favourably [59,62–64]. Ingroup favouritism also has consequences for people’s perceptions of others’ trustworthiness and their decisions to trust them [60,65–67]. From this point of view, shared group memberships are crucial for trust and cooperation [6,11].

There are different explanations for ingroup favouritism in the literature. We focus on social identity theory and bounded generalized reciprocity theory here. Social identity theory’s basic assumption is that humans derive their self-concept not only from their *individual*, but also from their *social* identity [68,69]. By assigning themselves and others to social categories, individuals gain “cognitive tools that segment, classify, and order the social environment, and thus enable the individual to undertake many forms of social action” (63, p. 16). Through this process of self-categorisation, individuals begin to identify with a group–their *ingroup*. Subsequently, they adopt the group’s identity, become emotionally attached to it and, eventually, their self-esteem gets linked to that of the group. Consequently, individuals seek to bolster their group’s image–and, thereby, their self-image–by drawing social comparisons to *outgroups* that shed a positive light on the ingroup [69]. This sequence of categorising, identifying and comparing eventually results in a bias for the ingroup, which manifests itself in the tendency to view attitudes and behaviours of ingroup members more favourably than those of outsiders [70].

An important alternative to the social identity explanation of ingroup bias is based on reciprocity. From this perspective, social identity theory has overlooked the fact that in the typical experimental tests of this theory, individuals are not only categorized in groups, but also exhibit a specific interdependence structure. In many experiments, subjects have to divide certain resources among ingroup and outgroup members and, thus, their individual outcome is dependent on the decisions of other participants. By removing this interdependence structure, for instance by giving parts of the experimental group a fixed individual outcome, ingroup bias disappears [71]. Thus, bounded generalized reciprocity theory assumes that ingroup bias is not based on social identity, but on self-interest. Participants maximize their own outcomes based on the assumption that ingroup members will reciprocate their own ingroup bias (group heuristic) [72]. In meta-analyses and cross-cultural studies, this perspective receives stronger empirical support than social identity theory [73,74]. However, with our research design we are only able to distinguish between the role of status and ingroup bias; we are not able to distinguish between different theoretical explanations of ingroup bias.

How ingroup favouritism moderates trust has received considerable attention in previous research–with mixed results. Banuri, Eckel and Wilson [75], for instance, implemented a trust game between inhabitants of different residential colleges at Rice University. They find an interaction effect with the costs of trust: there is a strong ingroup bias when favouring ingroup members is cost-free. However, there is only moderate ingroup favouritism when trusting is costly. Tan and Vogel [76] analysed ingroup bias in the context of trusting people from different religions. However, they find no evidence for ingroup bias. This is in line with other research on national or ethnic groups: some do observe ingroup favouritism [19], while others do not [17]. Some studies even discover *outgroup* favouritism [77] or discrimination against one particular group, even by its own members [18]. Another strand of research examines trust between members of “minimal groups” [69], i.e. based on artificial criteria with no real-life relevance. Güth, Levati and Ploner [78], for example, randomly assign participants to group “X” or group “Y”; here, the authors detect no effects of group membership on trust in the trust game. Similarly, Hargreaves Heap and Zizzo [79] split their participants into two meaningless groups at random: “red” and “blue”. They find no evidence for ingroup favouritism but, instead, moderate outgroup derogation.

From the theoretical considerations outlined above, we derive our final research hypothesis:

#### Ingroup hypothesis

*Individuals (i) perceive members of their ingroup as more trustworthy than members of the outgroup and (ii) behave more trustworthy towards members of their ingroup compared to outgroup members, irrespective of the cultural legitimacy or social status of each group*.

## Methods and data

We conducted an online experiment to investigate how group membership affects intra- and intergroup trust. In the following section, we provide a detailed description of the *trust game* paradigm, the *strategy vector method*, the sampling procedure and the resulting sample.

### The trust game

We implemented the trust game [80], which is the most simple abstract representation of real-life trust situations. In the trust game, two participants are paired. One takes the role of the trustor and the other the role of the trustee. In our implementation, both players are endowed with 20 Swiss Francs (CHF) (approx. USD 20). The sender (the trustor) can send any share–i.e., none, some, or all–of this endowment to the receiver (the trustee). The amount is then tripled and the receiver can decide to return anything between nothing and everything to the sender.

The logic of the game is as follows: if both players seek to maximise joint payoffs and an equal distribution, the sender should send everything and the receiver should reciprocate so that both receive half of the joint payoffs. More specifically, the sender invests the entire endowment of CHF 20 and the receiver returns CHF 40 (the receiver receives 3* CHF 20 + 20 (initial endowment) = CHF 80 and, by splitting it in half, both end up with CHF 40). However, if the sender follows this logic and the receiver is a rational egoist, she is tempted to exploit the sender’s trust and keep the entire CHF 80 (leaving the sender with CHF 0). Thus, the sender may be hesitant to invest a large proportion of her endowment and might only send some or none of it, foregoing a potentially greater payoff but, at the same time, protecting her initial endowment. Therefore, the sender’s decision is a measure of placing trust and the receiver’s decision of trustworthiness (reciprocating trust).

### The strategy vector method

To test our hypotheses, our implementation of the trust game includes information about the group membership of the trustee. Hence, trustors are asked to specify how much of their CHF 20 endowment they will send if they are paired with a classical music and folk music enthusiast, respectively. This is done by going through all possible combinations using the strategy vector method [81,82]. This method resembles factorial surveys (vignette experiments)–with the difference that decision are payoff-relevant: at the end of the experiment, each subject A is randomly assigned to either the role of sender or receiver and paired with another subject B. Their respective identities (folk or classic fan) and roles will determine each one’s actual payout.

**Fig 1:**
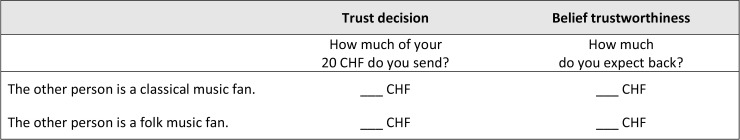
Trust decisions and beliefs of trustworthiness. The table shows how participants were asked for their investment decisions for the case that they were randomly allocated to the role of the sender (trustor). They are asked to make their decision in case they are matched with a classical music lover or a folk music lover. In addition, they are asked about their expected back transfers from each type of player they are matched with. The exact instructions are given in the text and the original screenshots from the online experiment are in the S1 File.

**Fig 2:**
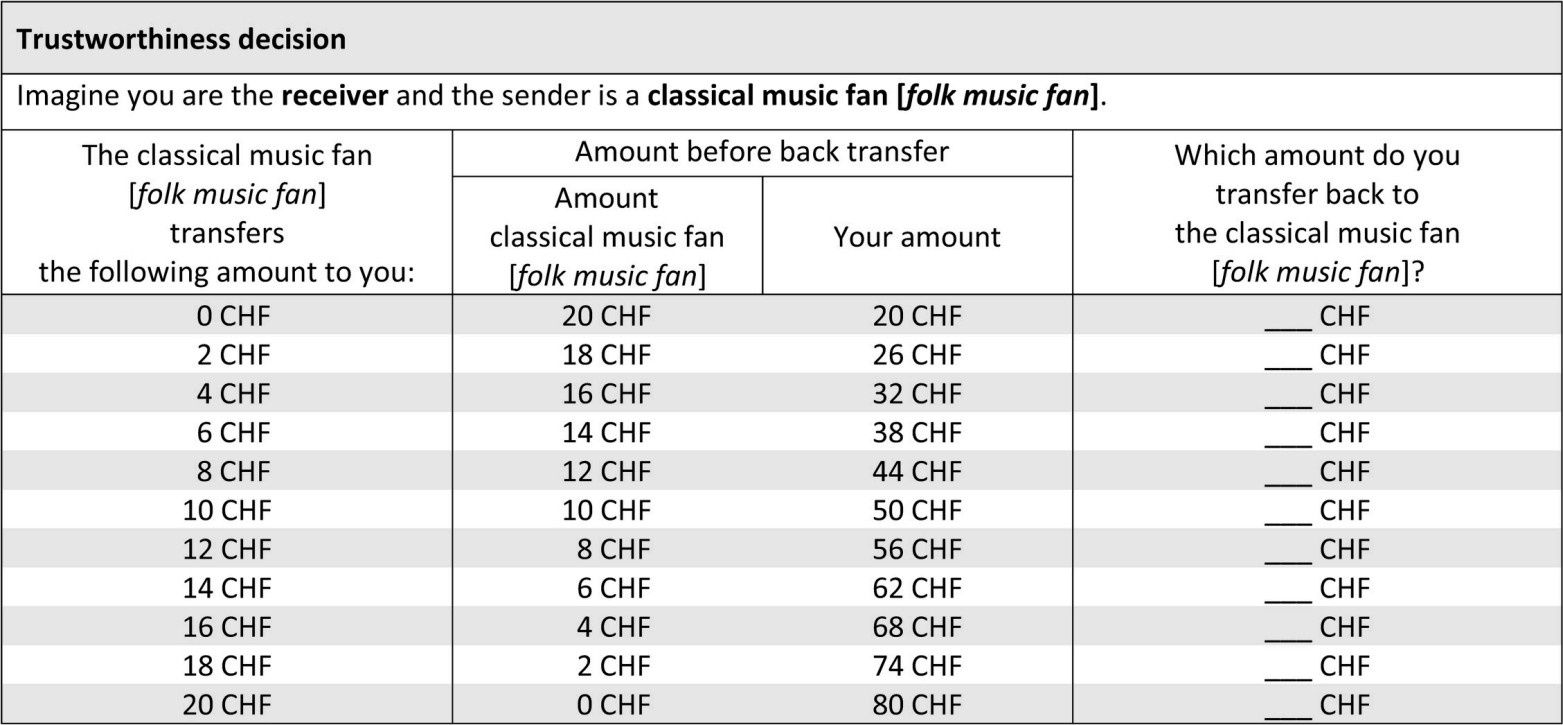
Trustworthiness decisions. The table shows how participants were asked for their back transfers for the case that they were randomly allocated to the role of the receiver (trustee). Participants received two separate screens–one for the case that they are randomly matched with a classical music lover and one for the case that they are randomly matched with a folk music lover. All else remains unchanged. These two variants are indicated with brackets and text in italics, i.e.: classical music fan [*folk music fan*]. The exact instructions are given in the text and the original screenshots from the online experiment are provided in the S1 File.

More specifically, the following procedure was employed: subjects were asked to state their decisions for each possible situation *before* being assigned to the role of sender or receiver. Thus, we asked all participants (a) “If you were the sender, how much money would you send to folk music lovers and classical music fans?”, (b) “If you were the sender, which amount would you expect to receive back from folk music lovers and classical music fans?” and (c) “If you were the receiver, how much money would you return–conditional upon the amount you received from the sender–to folk music lovers and classical music fans?”. The sequence of choices presented to participants during the game is illustrated in Figs 1 and 2.

Participants were informed that–*after* stating their decisions for all scenarios–they will be assigned to the role of the sender or receiver and paired with a classic- or folk-loving participant. All of these decisions are payoff-relevant since the pairing determines which of their choices is actually paid out. We can illustrate this using an example involving the previously mentioned subjects A and B. Assume A is a folk lover and assigned to the sender role and B is a classic fan in the receiver role. Then, the investment A stated for this particular situation–being the sender and paired with a classic fan–will be transferred to B. Consequently, A will receive the amount in return that B stated she would return if she received this particular investment from a folk lover. Each subject could decide to receive their entire earnings (via mail or bank transfer), which 40.2% of participants opted for, or to donate all or part of their earnings to a charity organisation (41.1% and 16.7% of participants, respectively). Average final earnings (irrespective of the decision to keep or donate the money) amounted to CHF 31.92.

To put it differently, these are not merely hypothetical situations (as in vignette experiments), but have real consequences in terms of gaining or losing money. In addition, the strategy vector method has the great advantage to yield substantially more data on subjects’ decisions regarding their trust decisions, trustworthiness and respective beliefs for all combinations of group memberships. Even though the strategy vector method might suffer from some shortcomings–e.g., mental overload or a lack of emotional involvement, i.e. “cold” decisions–, it is a valuable method yielding rich data. Rauhut and Winter [81] provide a detailed discussion of advantages and disadvantages in a variety of different applications. Brandts and Charness [83] review 29 empirical studies comparing the strategy vector method and the direct response method (only asking for decisions in one scenario). They find that the majority (16) of those studies finds no difference between the two methods, some (9) report mixed results and only a handful (4) do find differences. However, “in no case […] a treatment effect found with the strategy method is not observed with the direct-response method” (72, p. 375). There are studies that find smaller effect sizes in strategy vector experiments, potentially suggesting a cognitive overload for some but not all subjects. Thus, we argue that the strategy vector method produces potentially too conservative results, which have yet more statistical precision (smaller standard errors due to more decisions per subject). In addition, it allows a fine-grained analysis of the complete decision space, which makes it more valuable than data from direct response trust games. The elicitation of the trust decisions and beliefs about trustworthiness is illustrated in Fig 1.

The following instructions were given to subjects (translated from German):

“On this page, you can make your transfer decisions. Since you do not yet know if you will participate in this study in the role of the sender or the receiver, we ask you to state your decisions for both roles. First, you state your decisions as sender. Here, you have to indicate how much you send to each receiver group, i.e. to classical music fans and to folk music fans. Transfers can be made in steps of 2 (i.e. 0, 2, 4, …, 20 CHF). In the first row of the decision table, you state how much you would send if the receiver was a classical music lover. In the second row, you indicate how much you would send if the receiver was a folk music lover. In the case the receiver is a folk music fan, your decision for “folk music lovers” will come into effect. In addition, we are interested in your expectations concerning back transfer. Given your investment, how much do you expect to receive in return? Please indicate how much you except back given the other person is a classical music fan or a folk music fan in the corresponding row.”

The elicitation of trustworthiness decisions is illustrated in Fig 2.

The following instructions were given to subjects (translated from German):

“In the table below, you can enter your decisions regarding your back transfers in case you are assigned to the role of the receiver. Here, you have to indicate how much you would like to send back in case the sender is a classical music fan and in case the sender is a folk music fan. In the first column, you can see all possible investments the sender could choose from. Next to this column, you can see the resulting account balances given the senders investment choice. These are the account balances prior to your back transfer. Lastly, you see the column “Which amount do you transfer back to the classical music fan [*the folk music fan*]?” Here, you insert your back transfer decision. Since you do not yet know how much the other person invests, you have to choose for each possible investment which amount you would like to return. In the first row, you indicate how much you would like to return given the sender invested 0 CHF. In the second row you indicate how much you would like to return given the sender invested 2 CHF. It is essential that you state your back transfer decisions for all 11 possible investments. In each row, you can indicate any back transfer, i.e. 0, 1, 2, 3, … 80 CHF. Given the other person invests 4 CHF, the decision you made for “4 CHF” will come into effect; assuming the other person invests 10 CHF, the decision you made for “10 CHF” will come into effect; etc.”

### Sampling procedure and participants

We sampled persons who clearly identified as either folk music lovers (low-brow culture) or fans of classical music (high-brow culture). Therefore, the conditions specified in the literature on ingroup favouritism are clearly fulfilled. Participants were recruited via two different channels. First, we reached out through advertisements on media dedicated to classical music (e.g. *Radio Swiss Classic* newsletter, *classicpoint*.*ch* website) or folk music (e.g. *Alpenrose* magazine, *volksmusiknet*.*ch* website). Second, suitable candidates were selected from a representative online survey conducted by the Link Institute Lucerne based on their music preferences.

Both recruitment procedures yielded a sample of 102 participants. Our final sample comprises of 63 classical music fans and 39 folk music fans. Each subject made 26 statements about intended investments, expected back transfers and intended own back transfers conditional upon the received investment, which results in 2'652 decision clustered in 102 individuals.

The average age is 49.9; classical music fans are on average 49.4 years old and folk music fans 50.6. There are 52% women in our sample; among the classical music fans, 63.5% are female, among the folk music lovers 33.3%.

All aspects of the study design have been approved by the Ethics Committee of the Faculty of Arts at the University of Zurich. Potential subjects were invited through various platforms and self-selected into participation, thereby expressing their consent to partake in the study. Therefore, we opted to forego an additional form where participants explicitly signed their consent. Data was analysed anonymously.

## Results

In order to assess the *status perception hypothesis*, we analysed whether lovers of folk and classical music fans are perceived differently in terms of their social status. Participants were asked to state their impressions of the occupational prestige, economic success and educational levels of both folk music fans and classical music lovers on a scale from 1 to 5 (1 = *not at all* to 5 = *very*). Specifically, participants were asked to answer six separate questions (three regarding their perception of folk lovers, three with respect to their judgments of classic fans): (a) How prestigious are occupations of folk fans [*classic fans*] typically? (b) How economically successful are folk fans [*classic fans*]? (c) How educated are folk fans [*classic fans*]? Subjects rated classical music fans higher on each dimension. They assessed classical music lovers’ typical occupations as more prestigious compared to folk fans’ (average ratings 3.92 vs. 2.78, p = 0.000), judged classic enthusiasts as more economically successful (average ratings 3.85 vs. 3.00, p = 0.000) and as more highly educated (average ratings 4.19 vs. 2.89, p = 0.000). Folk and classic fans did not differ significantly in their ratings of these attributes of the two groups (detailed reports of differences between folk and classic lovers in the perception of status related attributes can be found in S1 Table in S2 File). Therefore, it is clearly established that classical music lovers are seen as of higher status by members of both groups. Bourdieu’s idea that classical music is a sign of high status is therefore corroborated.

In addition to perceived status differences, we examined actual status differences between the folk and classic fans in our sample in terms of their highest educational qualification (ranging from “basic secondary education” (1) to “doctorate” (7)). We focused on status in terms of education for practical reasons: first, we did not collect information on participants income; second, a large proportion of our sample indicated no (current) profession (n = 38, i.e. 37.25% of all participants), instead stating “job seeking” (n = 1), “housewife”(n = 2), “student” (n = 13), or “pensioner” (n = 22). Our analyses showed that there is a significant correlation between music preferences and education level (χ^2^(6) = 17.41, p = 0.008). More specifically, regressing music preferences on education level reveals that with each additional level of education, the probability of stating a preference for classical music (rather than folk music) rises by 9 percentage points (average marginal effect of education level, p = 0.001). Thus, classic and folk music lovers in our sample were not only perceived as of different social status, but actually did differ in this regard, at least with respect to educational attainment. While only *perceived* status differences are directly relevant to the status perception hypothesis as well as to a successful manipulation of status in the experiment, this finding further corroborates our claim that music preferences correlate with social status.

**Fig 3:**
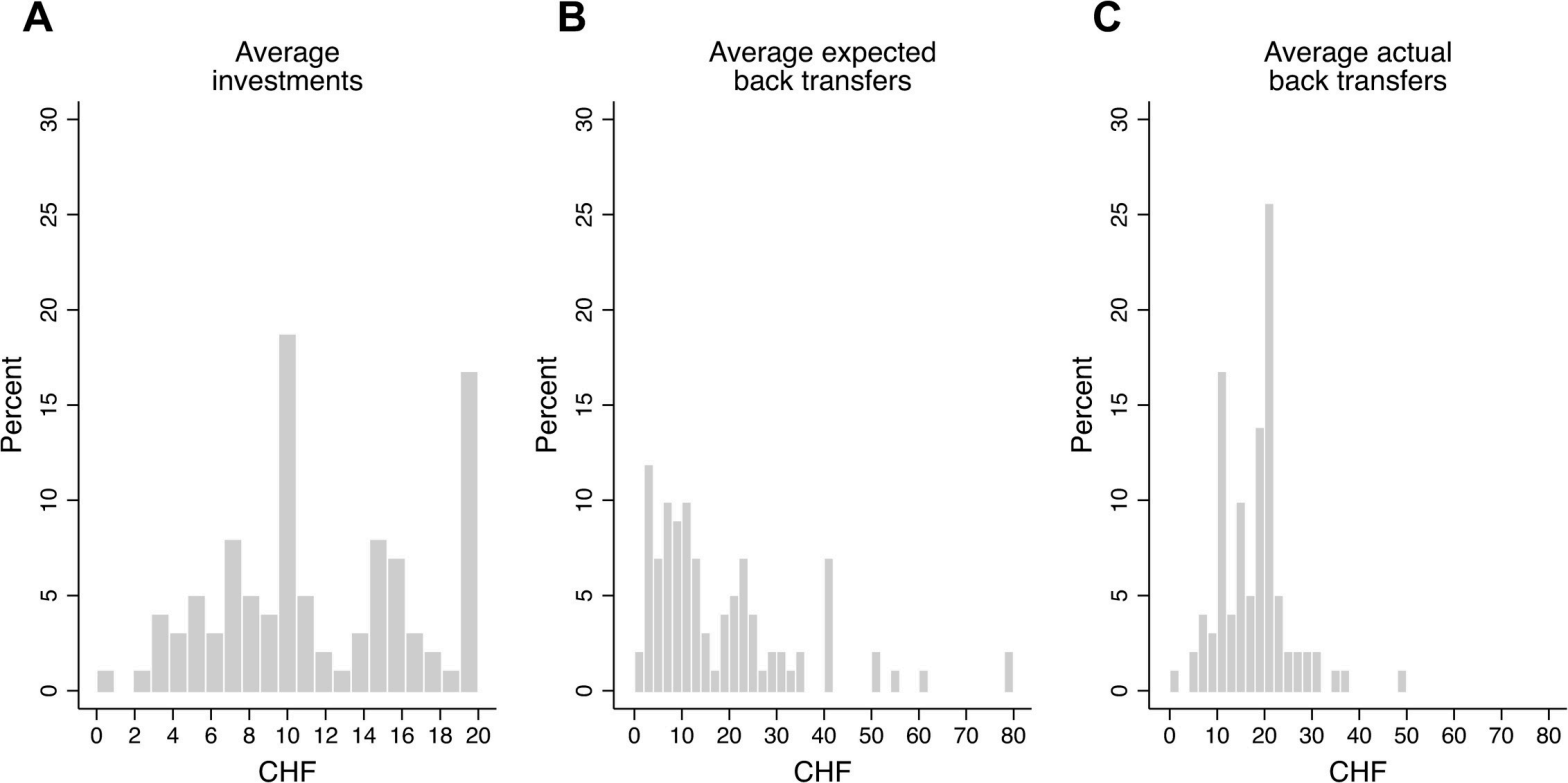
Trust, beliefs about trustworthiness and actual trustworthiness averaged over all groups. x-axes depict the amount (in CHF) participants stated they send (panel A), they expected back (panel B) and they transfer back (panel C); averaged over all back transfer decisions. Y-axes indicate percentage values.

In the next step, we explore descriptives about trust, beliefs about trustworthiness and actual trustworthiness. Fig 3 shows the distributions of investment decisions (panel A), expected back transfers (panel B) and back transfers averaged over the strategy vector method data (panel C) in Swiss Francs across all participants (n = 102).

Fig 3A illustrates a considerable heterogeneity in trust decisions. Decisions span the full range from no trust (investment of CHF 0) to full trust (investment of CHF 20) with an average of 11.79 (SD = 5.44). The majority of subjects invests either half (18.6%) or all (16.67%) of their endowment, expressing considerable trust into the trustee. Investments of zero occur only rarely, which implies that only a very small minority (1%) acts as rational egoists. Fig 3B shows that most people think they will receive at least some of their investments back. The average expected back transfer is 17.66 (SD = 15.90), two thirds of participants expect a return between CHF 4 and 30. None of the participants predict to receive nothing in return; there is also a small fraction (6%) of subjects who expect a hyper-fair back transfer of more than CHF 40 (i.e. more than half of the sum that is maximally available for both). As apparent in Fig 3C, returns of CHF 0 do indeed not occur at all, most transfers (2/3) range between CHF 10 and 21 with a mean of 17.46 (SD = 7.15).

**Fig 4:**
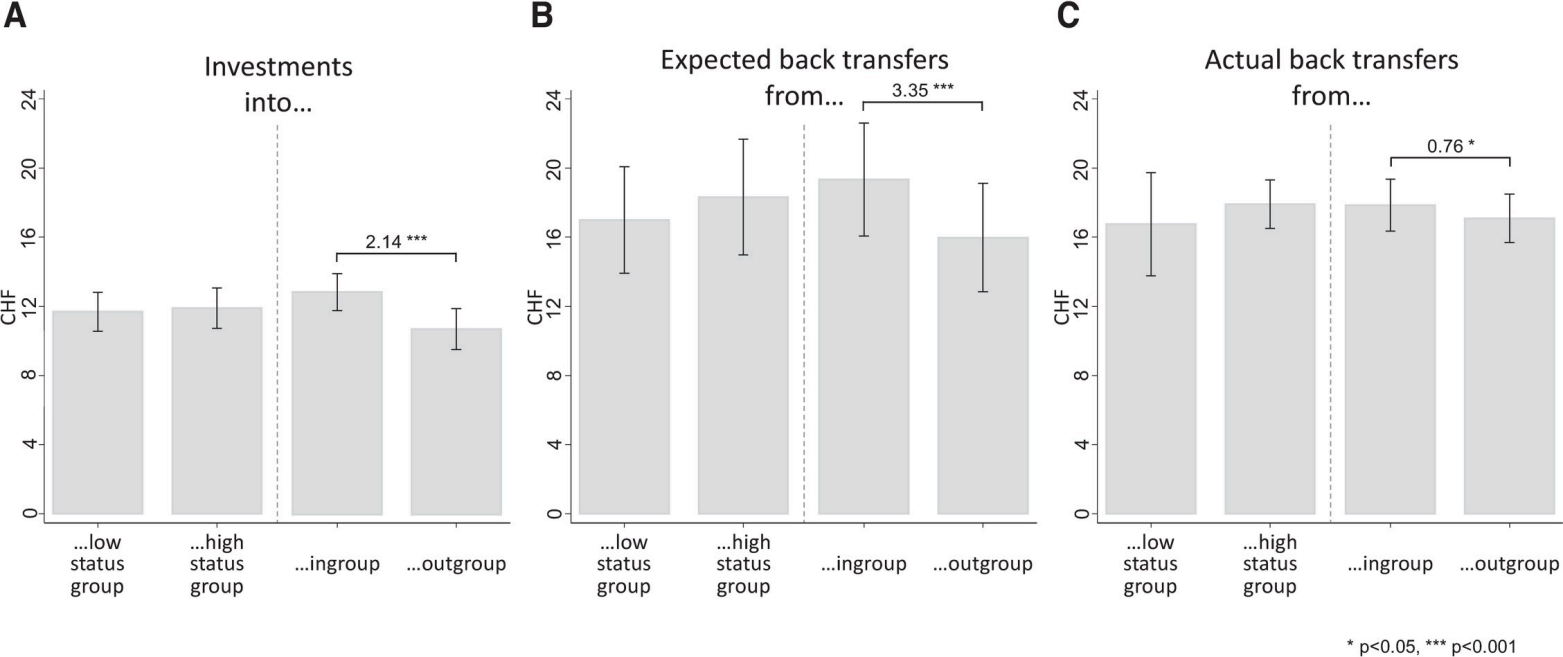
Investments, expected and actual back transfers: Low- vs. high-status group and in- vs. outgroup. x-axes depict the amount of CHF participants stated they would invest (panel A), they expected back (panel B) and they would transfer back (panel C; mean over all back transfer decisions) *conditional upon their counterpart’s group membership*; for each panel, the left part compares decisions when paired with a low-status vs. high-status partner, the right part decisions when paired with an in- vs. an outgroup partner; significant differences are indicated above the bars; * p<0.05, ** p<0.01, *** p<0.001.

We next turn to testing the *status* and the *ingroup hypothesis*. Fig 4 presents an overview of subjects’ decisions contingent on whom they are matched with (classic or folk). It shows the effect of group membership on trust, beliefs about trustworthiness and actual trustworthiness. To examine these effects, we calculated the differences between the means of (a) investments into classic fans and investments into folk enthusiasts, (b) expected back transfers from classic lovers and expected back transfers from folk fans and (c) actual back transfers from classic types and actual back transfers from folk lovers (details on group means and differences can also be found in S2 Table in S2 File). To test for statistical significance of these differences, we performed t-tests; in addition, we report Cohen’s d as a measure for effect sizes.

If high-brow cultural preferences indeed signal social status and thus trustworthiness, we should observe larger investments into the high-status group (i.e. classic fans) as well as higher expected and actual back transfers from this group, irrespectively of participants’ own music preferences. If, however, trust and trustworthiness hinge upon *shared* group membership (that is, if there is an ingroup bias), we should observe greater investments into one’s ingroup as well as larger expected and actual returns from the in- than the outgroup.

Fig 4A depicts mean investment decisions. The left part of Fig 4A reveals investment choices into receivers belonging to the low-status and the high-status group, independently of participants’ own music preferences. The difference between investments into folk and classic enthusiasts is small (CHF 0.22) and statistically insignificant (p = 0.616, d = 0.05), which rejects the status hypothesis.

The right part of Fig 4A shows investments into recipients belonging to one’s ingroup compared to those belonging to the outgroup. In other words, ingroup refers to cases where classic types invest into other classic types or folk types who invest into other folk types. Outgroup refers to cases where classic types invest into folk or folk types invest into classic. Here, we find clear support for the ingroup hypothesis: on average, subjects invest CHF 2.14 more if the receiver shares their own music taste (p = 0.000, d = 0.57).

Fig 4B shows expectations about back transfers (i.e. beliefs about trustworthiness). Expected returns from the folk-lover group compared to those from the classic fans group are displayed on the left. The difference between expected back transfers from folk and classic fans is slightly larger than for investments. On average, subjects expect to receive CHF 1.33 more back from classical music lovers than from folk music lovers. This difference, however, is not statistically significant at the 5%-level (p = 0.095, d = 0.17). Again, these findings reject the status hypothesis. The right half of Fig 4B reveals expected returns from in- versus outgroup members. Here, we see a much larger gap. When paired with an ingroup member, participants expect CHF 3.35 more in return than when matched with an outgroup-receiver. This difference is statistically significant (p = 0.000, d = 0.45). This corroborates again the ingroup hypothesis.

Fig 4C shows average actual back transfers. These are computed as mean back transfers averaged over all received transfer possibilities. Back transfers from the high-status group exceed those from the low-status group by CHF 1.16 (left). This difference is, however, not significant (p = 0. 0.429, d = 0.16). Actual back transfers from the ingroup exceed those from the outgroup by CHF 0.76. This is statistically significant (p = 0.047, d = 0.20). This finding is the third evidence supporting the ingroup hypothesis and rejecting the status hypothesis.

We additionally examined if these findings are driven by group-level patterns, i.e. if there are systematic differences between the choices made by folk and classic fans. We thus investigated if the differences we find for investments, expected back transfers and average actual back transfers in favour of the ingroup can also be observed when folk or classic oriented participants are considered separately. This is indeed the case. As above, we used bivariate mean comparisons and performed t-tests to test for statistical significance of any differences. We find that folk music fans invest CHF 2.51 (p = 0.001, d = 0.57) more into the ingroup than the outgroup, expect CHF 2.64 (p = 0.005, d = 0.48) more in return from the ingroup and send an additional CHF 1.87 (p = 0.040, d = 0.34) back when paired with an ingroup member (compared to an outgroup member). Classic fans also exhibit a pronounced ingroup bias, investing CHF 1.90 (p = 0.000, d = 0.57) more and assuming higher back transfers (+ CHF 3.79, p = 0.001, d = 0.45) when matched with a fellow classic lover; in terms of actual back transfers, however, there is no ingroup bias (+ CHF 0.08, p = 0.763, d = 0.04) in this group.

It should be noted that the differences compared above are not normally distributed. We believe that this does not affect the results in the present case: analyses rely on a sufficiently large sample size which ensures that the tests have sufficient statistical power and yield robust results even if the normality condition is not satisfied [84–86]. We nevertheless performed additional nonparametric tests to solidify the claims regarding statistical (in)significance of results. We complemented each t-test with a Wilcoxon matched-pairs signed-ranks tests as well as sign tests of matched pairs to forestall any doubt asymmetries in the distributions might cast on the results of the Wilcoxon matched-pairs signed-ranks test. Results confirm the conclusions inferred from the t-tests: in each instance, the test statistics of all tests correspond in terms of whether or not to reject the null hypothesis (detailed results of all tests can be found in S3 Table in S2 File).

In sum, we find that–*overall*–subjects trust folk fans and classic lovers to similar degrees and that they do not perceive the latter group as more trustworthy. Thus, there is–in contrast to the status proposition–no signalling effect of cultural consumption on trust. Instead, we find strong evidence for ingroup-based trust and more optimistic beliefs about back transfers from ingroup members. However, *actual* back transfers are much less group-biased: here, we only observe ingroup favouritism among folk music fans, but not among classical music lovers.

Taken together, these results refute the status hypothesis and lend support to the ingroup hypothesis. Our findings show that people tend to base their trust decisions on shared group membership and assume that ingroup members are more trustworthy. However, the observation that actual behaviour–that is, back transfers–is much less group-biased suggests that people hold unjustified stereotypes: trust is largely reciprocated independently of trustees’ group memberships. Thus, in a nutshell, classic trusts classic and folk trusts folk but this bias works–at least for classic fans–through false beliefs about group-based trustworthiness.

**Fig 5:**
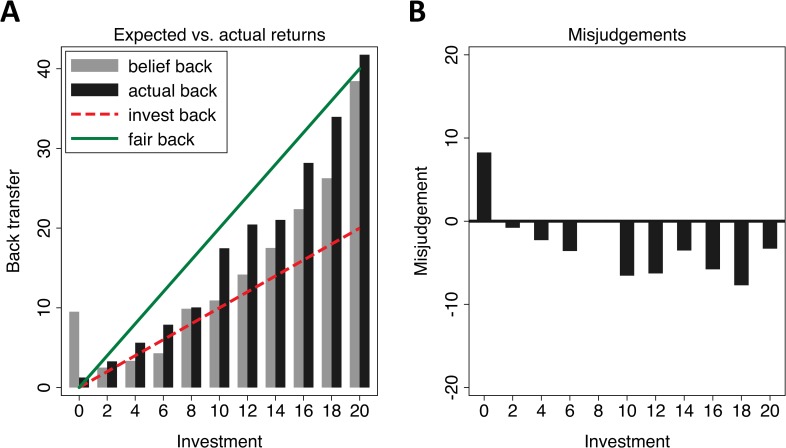
Expected vs. actual returns. Panel A: average *expected* back transfers vs. *actual* back transfers (y-axis) for all possible received investments (x-axis). The green solid line refers to the minimum back transfer such that senders receive what they have sent. The dashed red line refers to the respective amount, where the back transfer represents an equal split of joint earnings. The area in between the solid red and the dashed green line refers, therefore, to the space where the minimum marks sending decisions without losses and the maximum refers to what can be expected from a maximally prosocial (i.e. fully inequality averse) trustee. Panel B refers to the difference between expected and actual back transfers.

We now take a closer look at the discrepancy between beliefs and actual behaviour. Fig 5 compares expected back transfers and actual back transfers across all interactions for all participants and for the two groups separately.

In the left panel, average *expected* back transfers (black bars) and the corresponding *actual* back transfers (grey bars) are compared for all possible received investments from CHF 0 to 20 (x-axis). The dashed red lines refer to the minimum amount that has to be transferred so that senders receive at least their investments in return. The solid green lines refer to a fully equal split, i.e. the amount that can be expected from a maximally prosocial, fully inequality-averse trustee. In other words, the area in between both lines refers to back transfers where senders do not experience losses (minimum) up to the cases where they receive a fully equal split (maximum). The right panel illustrates the extent of false beliefs (mismatches between beliefs and outcomes). Downward (upward) deviations imply an underestimation (overestimation) of back transfers.

Fig 5 shows that back transfers exceed investments for all possible decisions except those of CHF 0; thus, every potential investment results (on average) in a positive payoff for the sender. *Over*estimations of back transfers only occur at the fringes of the range of possible investments in the extreme cases of investing nothing or everything: only those who invested none of the endowment tend to expect much greater back transfers than those who sent only a share or all of their initial CHF 20. Additionally, the results show that, on average, participants are less trusting than what would be rational given the average back transfers. This result holds irrespectively of group membership. For investments between CHF 2 and CHF 20, subjects largely misjudge the returns they will gain given the amount they invested and, therefore, both groups have false beliefs about the trustworthiness of their interaction partners.

In a next step, we examine if these systematic misjudgements are also affected by ingroup biases. To this end, we compare the difference between the back transfers participants *expect* for their investment and the amount they actually do *receive* in return. We make this comparison for trust game encounters of members of the same group (ingroup encounters) as well as of members of different groups (outgroup encounters). Specifically, we compute the difference between expected back transfers and average actual back transfers for each particular investment level for both ingroup and outgroup encounters. We then estimate the effect of an ingroup encounter (dummy variable = 1 for ingroup encounters, = 0 for outgroup encounters) on the extent of the gap between expectation and actual back transfers. In other words, we analyse if interacting with someone from one’s own group reduces the divergence between expectations and actual eventual returns while holding the amount one invested constant. We employ a linear regression model with clustered standard errors and include the difference between expected back transfers and average actual back transfers for each investment level as the dependent variable, the ingroup-encounter dummy as our main independent variable and a control for investment levels. We find that there is a substantial difference with regard to the accuracy of beliefs in ingroup versus outgroup encounters. The coefficient of the ingroup-encounter-dummy is negative (-1.92) and statistically significant (p = 0.011; detailed regression results are provided in S4 Table in S2 File). This result implies that the misjudgement is less pronounced when a participant is matched with an ingroup member and, thus, that shared group membership does improve predictions about one’s interaction partner’s trustworthiness. From this regression and the analysis above, it follows that actors are more optimistic and better informed about the trustworthiness of ingroup than outgroup members.

## Conclusion

This article set out to explore how group membership affects trust and trustworthiness. We contrasted two theoretical mechanisms. Individuals may place more trust in persons signalling high social status via a preference for classical music or they may trust people from their own group to a greater extent. We operationalised group membership by musical tastes, i.e. high- vs. low-brow cultural consumption. Our evidence shows that lovers of classical music are perceived as of higher social status compared to fans of folk music–by all participants. However, concerning the receiving and giving of trust, our experimental data rejects status effects and supports ingroup favouritism. Subjects trust folk fans and classic lovers to similar degrees and do not perceive the latter group as more trustworthy. This results is somewhat at odds with findings of previous studies employing the same experimental paradigm, whose results largely do demonstrate that persons of higher social status receive more trust. This discrepancy between prior work and the present study might be due to our operationalisation of social status via musical preferences. While the majority of previous studies manipulated status via very visible, straightforward indicators–such as living in a region commonly known as wealthy or disadvantaged [15,29], being member of a certain caste [28] or known income differences [16,30]–, our manipulation may have been more subtle. Our manipulation check clearly shows that knowledge about persons’ musical preferences does indeed evoke a certain perception of their status. However, it appears plausible that the association of musical tastes with status might happen on a less conscious level than that of income with status and, thus, might shape interactions to a lesser extent. This is a question that can only be answered empirically and may be a fruitful direction for future research.

Participants do, however, place significantly more trust in members of their own group and expect them to be more trustworthy. This expectation is shown to be partially unwarranted. *Actual* back transfers are much less group-biased: here, we only observe ingroup favouritism among folk music fans, but not among classical music lovers. Thus, when it comes to reciprocating trust, the bias for one’s ingroup is much less pronounced, which suggests that ingroup bias is, at least in part, driven by false beliefs. However, it is difficult to generalize from our example, since in other cases ingroup preferences may be based on true beliefs. This seems a very important question for further research.

Interestingly, we find that ingroup favouritism is most consistent among low-status (folk-music loving) participants. In this group, we observe strong ingroup favouritism across all trust-measures. This result suggests that the bias for ‘insiders’ is most pronounced in the low-status group, while the high-status group demonstrates a greater tolerance of ‘outsiders’. This implication of our findings is somewhat in line with evidence from research on prejudice and discrimination. In this field of study, it has repeatedly been shown that individuals with low socioeconomic status tend to be less accepting of members of various outgroups such as immigrants [87], sexual minorities [88] or the homeless [89].

We highlight two implications of our results for social theory: firstly, with respect to Bourdieu’s theory of social classes and “legitimate” culture and, secondly, concerning signalling theory.

Regarding Bourdieu’s theory of class and “legitimate” culture, it is indeed the case that–as hypothesized by Bourdieu–lovers of classical music are perceived as being of higher status than fans of folk music. This is, also supporting his theoretical assumptions, true for members of both groups in our study. Thus, not only classical music lovers perceive themselves as high in status and folk-music fans as lower in status, the same is true for folk music fans. As theorized by Bourdieu, engagement in high-brow culture is a sign of high status, which is perceived as such throughout different groups of the society. However, Bourdieu went further in his theoretical discussions and assumed that high-brow culture is a form of “legitimate” culture in society because it is associated with persons of high social status and power and institutionalized in school curricula and cultural policy. According to Bourdieu, this status of “legitimate” culture should produce awe, reverence and trust in all members of society. Our results did not support the hypothesis that high-status individuals are trusted to a greater extent. Classical music lovers were not perceived as more trustworthy than folk music fans. Therefore, our results do not support the idea that contemporary societies exhibit something like a hierarchy of cultural tastes, where some tastes are considered as “legitimate” and dominant, thus engendering trust. Different taste groups are able to recognise their differences, but adherents of other tastes do no invest more trust in those who participate in high-brow culture [51,90].

Signalling theory assumes that, in a sorting equilibrium, the honest signaller has lower costs in producing a certain signal compared to a dishonest signaller. Thus, a certain signal–like, in our case, musical preferences–should reflect underlying objective differences–such as different levels of trustworthiness–between the signallers. In a sorting equilibrium, the trustees have a *true* belief that a certain signal is a *true* signal of trustworthiness. However, we found that respondents had clear preferences for trusting their ingroup members. In our study, these preferences have been shown to be partially based on false beliefs–at least among members of the high-status group. This is somewhat at odds with findings from previous research demonstrating that expecting greater prosociality from one’s ingroup is well-justified. Evidence suggests that shared group memberships increase empathy and one’s sense of (ingroup) responsibility [91] and that even infants already expect more support and helping behaviour in interactions between persons belonging to the same group than between individuals belonging to different groups [92]. Our own results that trust-related ingroup bias is to some extent unwarranted could be explained in two ways. Firstly, we can assume that our results are not based on a sorting equilibrium, but are simply a snapshot of transient interactions. Since both of our groups are likely to have previously interacted with members of their own ingroup (because interpersonal networks are strongly shaped by cultural preferences), this explanation is rather unlikely though [93]. A second possible explanation could be that some false beliefs are highly stable, in spite of contradictory evidence, as, for instance, suggested by the continuing success of cons. Thus, future research on signalling should take the possibility of stable false beliefs much more into account.

Another aspect that future research should take into account is social status at the “trusting end”: in the present study, the main research interest was in whether high-status individuals would be perceived as more trustworthy and if they would be more likely to reciprocate the trust invested in them (i.e., social status at the “trust-receiving” end). A question that was not of central interest here was whether persons of high social status would also be more inclined to place their own trust in others. Research has shown that (induced) high social status can indeed lead actors to perceive their interaction partners as more benevolent and, consequently, to be more trusting [94]. Given Bourdieu’s [13] arguments that persons adhering to cultural tastes signalling high social status are treated with deference by others, they may be more inclined to assume that others have good intentions. Therefore, it appears plausible that, also in the context of culture-based groups, social status might shape intra- and intergroup trust in terms of one’s propensity to grant trust to others. While preliminary analyses based on our data do not suggest that members of high-status groups tend to be more trusting than those of lower-status groups (see S5 Table in S2 File), a more thorough exploration of this proposition might still present an interesting and fruitful avenue for future research.

Aside from their scientific value, our findings have implications with respect to improving the establishment of trust in interactions between strangers. On the one hand, the results of our study imply that interactions between members of different groups can be biased by false expectations about uncooperative behaviour of outgroup members. Raising awareness for this “statistical discrimination by false beliefs” may be a first step to ameliorating cooperation between unacquainted individuals. On the other hand, our results support the well-established assumption that a common culture enhances trust and cooperation [12] and are relevant in many contexts where trust has to be newly established on a regular basis–for example, in work contexts where employees collaborate in frequently changing team configurations.

However, there are some limitations to our research design. Firstly, subjects were recruited via advertisements (websites, magazines) or contacted directly after they participated in a culture survey and could sign up for the experiment. Therefore, we have a self-selected sample of persons clearly identifying as classical music lovers or as folk music fans. This might bias the results towards ingroup favouritism. However, this is contradicted by the fact that both groups have rather realistic assessments of their own group’s social status. Secondly, the strategy vector method has sometimes been criticized for potentially leading to mental overload or for suffering from a lack of emotional involvement (“cold” decisions). Despite this criticism, we argue that the strategy method produces more reliable data than survey questions or vignette studies–since decisions are directly payoff-relevant–and also yields a richer dataset than traditional (lab or online) experiments employing the direct-response method. Lastly, Switzerland–unlike many of its neighbours–lacks a strong *own* tradition of high-brow culture. Even though the cultural discourse in Switzerland is quite traditional, high-brow culture has been less dominant in its society than in those of other European countries because of the absence of princely courts (e.g., France, Germany, Italy); there even prevails a kind of sturdy resistance towards its elitism in the common population [44]. Thus, the effects of participation in high-brow culture might be less pronounced than in societies with a strong tradition of such cultural practices. Thus, there should be some replications of our study in countries, which have a stronger orientation towards high-brow culture. This would clarify the debate on the relevance of status-effects versus ingroup preferences further.

## Supporting information

S1 File Original instructions and questions. (DOCX)

S2 File Detailed tabularised report of analyses reported in the main text. (DOCX)
