# Supplementary material for: Is participation in high-status culture a signal of trustworthiness?
Source: PLoS One. 2020 May 5;15(5):e0232674. doi: 10.1371/journal.pone.0232674 (PMC7199962; doi:10.1371/journal.pone.0232674)
Supplement: S1 File — (DOCX) [file pone.0232674.s001.docx]

**S1 Original instructions and questions**

**S1.1 Information about the study provided to participants (translated from German)**

Participants were provided with information about the study at two points: first, in the advertisement for the study, which gave basic information about the content and the aim of the experiment. If participants decided to follow the link redirecting them to the study, they reached a second set of information materials (on the first screen they were presented with). The following information (translated from German) was provided:

Stage 1: Information in study advertisement

Scientific studies have shown that musical preferences affect our lives in a wide range of areas: starting with the choice of friends and ending with our eating habits. At the University of Zurich, an online experiment is now investigating whether the trust we place in other people has something to do with our taste in music. Participants receive real money in the experiment. Participants can exchange this money with other participants. The more you trust others and the more others trust you, the more money you make. The experiment can be reached via the following link: [link]. We would be very happy about your participation.

Stage 2: Information provided at the beginning of the study

Welcome to our scientific study on trust. The study is conducted by the Institute of Sociology at the University of Zurich. We would be very pleased about your participation. We provide you with an endowment of 20 Swiss Francs for this study. You can use this amount in a decision experiment to increase your profit. At the end of the study, you can let us know if you would like to receive your prize by mail or bank transfer.

By participating in the study you make a valuable contribution to scientific research! In addition, you have the opportunity to earn money in a short time.

The study consists of two parts, which you can find on the following pages:

1. A decision experiment.

2. A questionnaire.

What should you do?

1. Please first read the description of the decision section on the following pages.

2. Then complete the decision experiment completely.

3. Then complete the questionnaire completely.

The data of this study will be used in anonymous form and for scientific purposes only. The documents for this study were submitted to the Ethics Committee of the Faculty of Arts at the University of Zurich for review and judged by the Ethics Committee free from potential ethical concerns.

If you are interested in the results of the study, please provide a contact address at the end of the questionnaire. The address will of course be kept separate from your details in the questionnaire. Also, the information you provide to receive your prize will be treated separately from the data in the study.

If you have any questions about the study (for example, to complete the decision sheet), we will be happy to answer your queries by phone [phone number] or email [email address]. Do not hesitate to contact us!

**S1.2 Instructions and questions trust experiment (in German)**

**
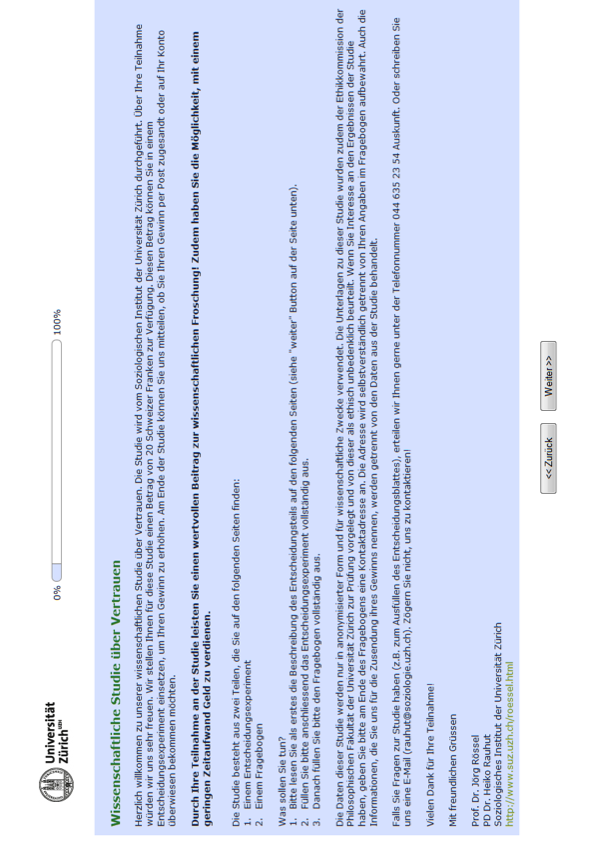
**

**Figure S1 Introduction of the study** This screenshot shows how participants were introduced to the study in general.

**
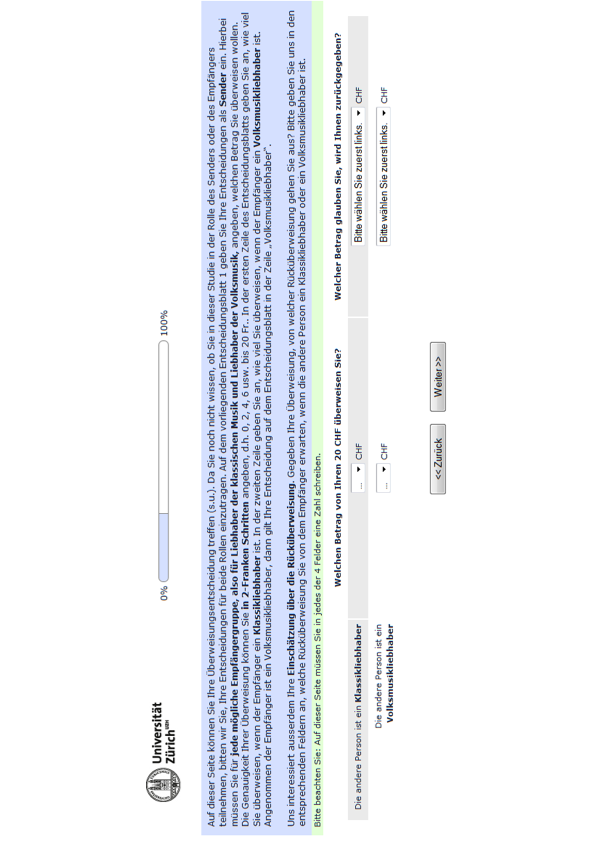
**

**Figure S2 Instructions for the Trust Game Part 1: General information and examples** This screenshot shows how participants were introduced to the game and which examples were provided to increase their understanding of the game.


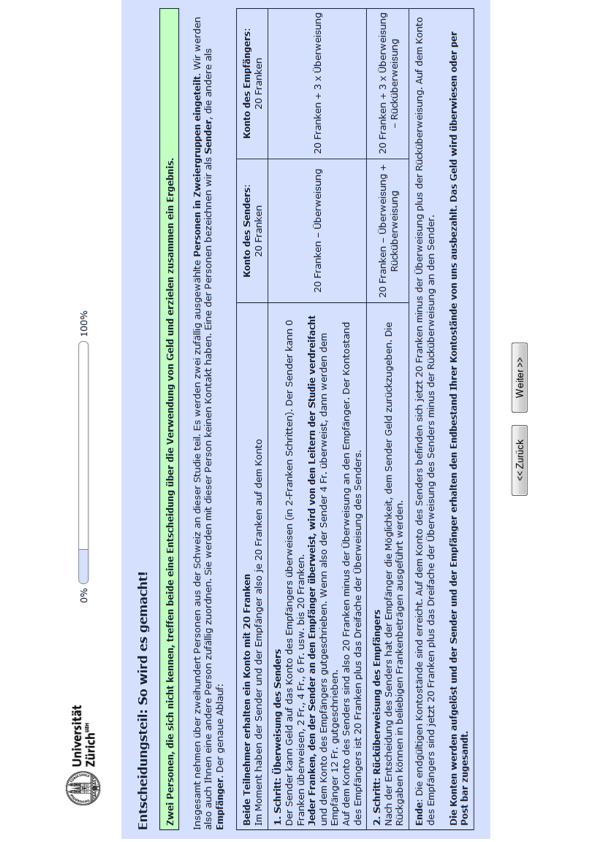

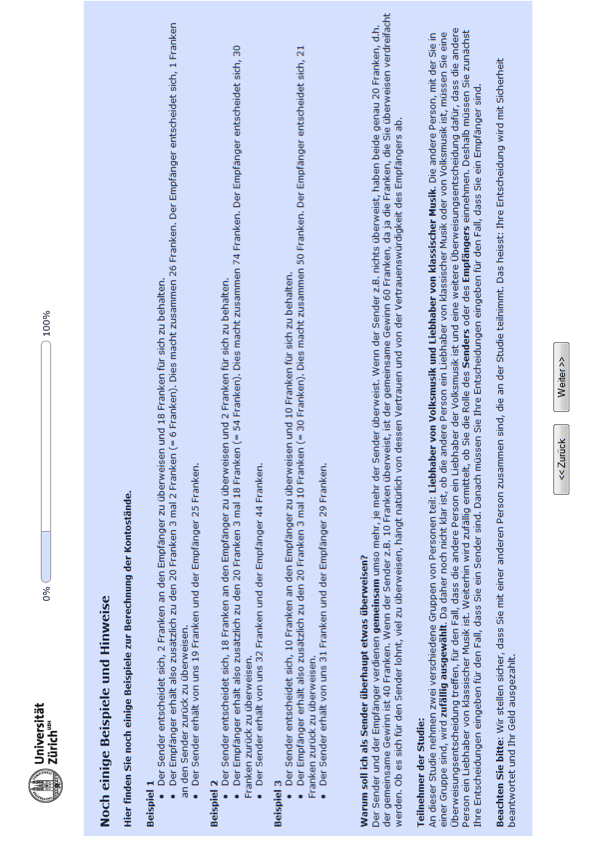


**Figure S3 Instructions for the Trust Game Part 2: Trust decisions and beliefs of trustworthiness** This screenshot shows how participants were asked for their investment decisions for the case that they were randomly allocated to the role of the sender (trustor). They are asked to make their decision in case they are matched with a classical music lover or a folk music lover. In addition, they are asked about their expected back transfers from each type of player they are matched with.

**Figure S4 Instructions for the Trust Game Part 3: Trustworthiness decisions** This screenshot shows how participants were asked for their back transfers for the case that they were randomly allocated to the role of the receiver (trustee). These screens were displayed separately: First, the one for the case that participants are randomly matched with a classical music lover. Second, the one for the case that participants are randomly matched with a folk music lover. All else remains unchanged.


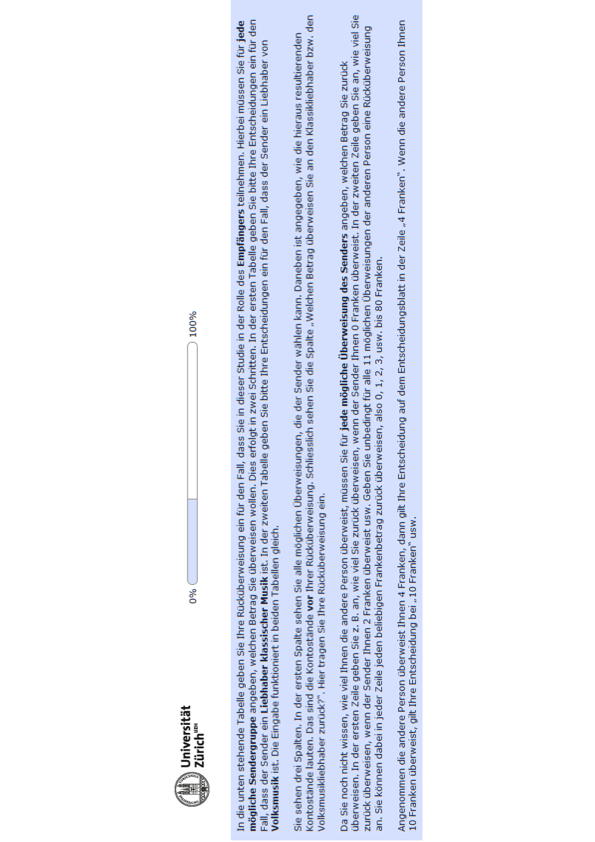

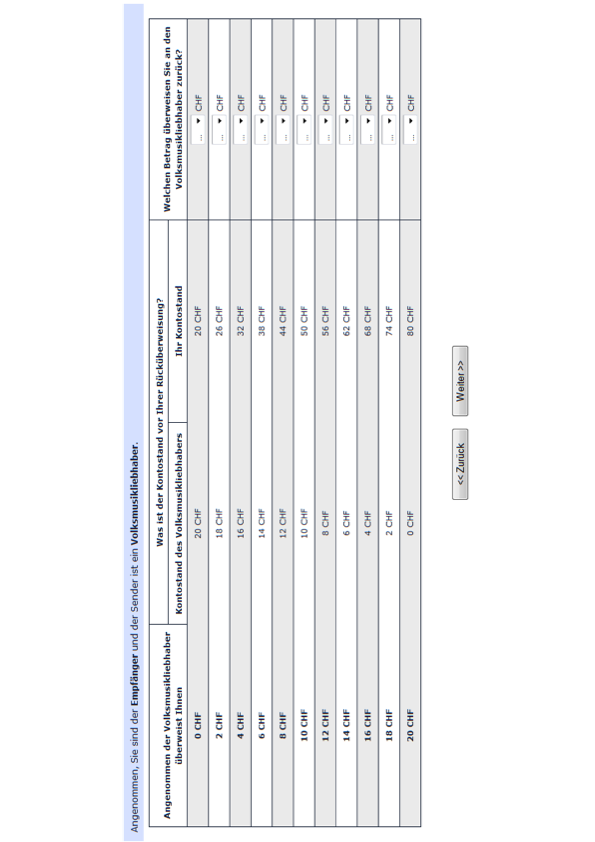

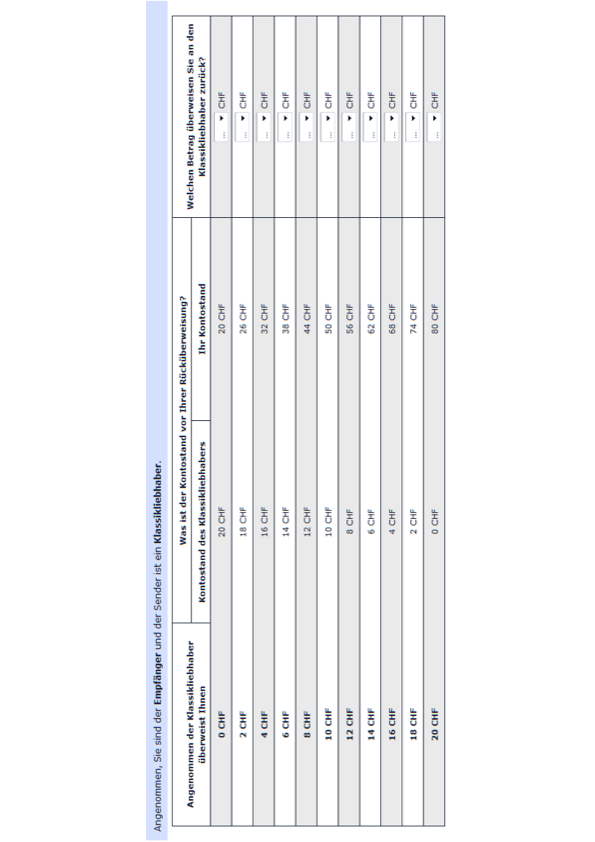


**S1.3 Questions assessing the perception of the status (occupation, economic success and education levels) of folk and classic fans (in German)**

| Wie hoch ist das **Ansehen der Berufe** von Klassikliebhabern typischerweise? | Sehr  gering | ❑ ❑ ❑ ❑ ❑  1 2 3 4 5 | Sehr hoch |
| --- | --- | --- | --- |
| Wie **wirtschaftlich** **erfolgreich** sind Klassikliebhaber? | Sehr  gering | ❑ ❑ ❑ ❑ ❑  1 2 3 4 5 | Sehr hoch |
| Wie **gebildet** sind Klassikliebhaber? | Sehr  gering | ❑ ❑ ❑ ❑ ❑  1 2 3 4 5 | Sehr hoch |

| Wie hoch ist das **Ansehen der Berufe** von Volksmusikliebhabern typischerweise? | Sehr gering | ❑ ❑ ❑ ❑ ❑  1 2 3 4 5 | Sehr hoch |
| --- | --- | --- | --- |
| Wie **wirtschaftlich** **erfolgreich** sind Volksmusikliebhaber? | Sehr gering | ❑ ❑ ❑ ❑ ❑  1 2 3 4 5 | Sehr hoch |
| Wie **gebildet** sind Volksmusikliebhaber? | Sehr gering | ❑ ❑ ❑ ❑ ❑  1 2 3 4 5 | Sehr hoch |
