# Supplementary material for: Is participation in high-status culture a signal of trustworthiness?
Source: PLoS One. 2020 May 5;15(5):e0232674. doi: 10.1371/journal.pone.0232674 (PMC7199962; doi:10.1371/journal.pone.0232674)
Supplement: S2 File — (DOCX) [file pone.0232674.s002.docx]

**S2. Detailed tabularised report of analyses reported in the main text**

**Table S1** **Absolute differences between folk and classic lovers in the perception of status related attributes**

| Perception of | Mean  among folk fans | Mean  among classic fans | Absolute  difference | p |
| --- | --- | --- | --- | --- |
| Occupational prestige of folk lovers | 2.71 | 2.81 | 0.10 | 0.5743 |
| Occupational prestige of classic lovers | 3.92 | 3.92 | 0.00 | 0.9884 |
| Economic success  of folk fans | 3.00 | 3.00 | 0.00 | 1.0000 |
| Economic success  of classic fans | 3.90 | 3.83 | 0.07 | 0.6561 |
| Educational levels  of folk fans | 2.92 | 2.87 | 0.05 | 0.7334 |
| Educational levels  of classic fans | 4.15 | 4.21 | 0.05 | 0.6965 |
| n | 39 | 63 | 102 |  |

*Perception of the social status of persons with a preference for folk / classical music by folk / classic lovers. Means refer to statements about perceived occupational prestige, economic success and educational levels on a scale from 1 to 5 (1=not at all to 5=very). Perceptions were elicited through six questions: (1[4]) How prestigious are occupations of folk fans [classic fans] typically? (2[5]) How economically successful are folk fans [classic fans]? (3[6])*

**Table S2** **Detailed** **group means and differences in investments, expected and actual back transfers**

|  | All  participants | Folk  music fans | Classical music fans |
| --- | --- | --- | --- |
| (1a) Investments into Low- vs. High-status group |  |  |  |
| Average investment into folk music fans | 11.67 | 13.03 | 10.86 |
| Average investment into classical music fans | 11.90 | 10.51 | 12.76 |
| Difference | 0.22 | 2.51 | 1.90 |
| *p* | 0.616 | 0.001 | 0.000 |
| (1b) Investments into Ingroup Vs. Outgroup |  |  |  |
| Average investment into ingroup | 12.86 | 13.03 | 12.76 |
| Average investment into outgroup | 10.73 | 10.51 | 10.86 |
| Difference | 2.14 | 2.51 | + 1.90 |
| *p* | 0.000 | 0.001 | 0.000 |
| (2a) Exp. back transfers From Low- vs. High-status group |  |  |  |
| Average exp. back transfers from folk music fans | 16.99 | 19.05 | 15.71 |
| Average exp. back transfers from classical music fans | 18.32 | 16.41 | 19.51 |
| Difference | 1.33 | 2.64 | 3.79 |
| *p* | 0.095 | 0.005 | 0.001 |
| (2b) Exp. back transfers From Ingroup Vs. Outgroup |  |  |  |
| Average exp. back transfers from ingroup | 19.33 | 19.05 | 19.51 |
| Average exp. back transfers from outgroup | 15.98 | 16.41 | 15.71 |
| Difference | 3.35 | 2.64 | 3.79 |
| *p* | 0.000 | 0.005 | 0.001 |
| (3) Actual back transfers |  |  |  |
| Actual back transfers from folk music fans | 16.75 | 17.69 | 17.87 |
| Actual back transfers from classical music fans | 17.91 | 15.81 | 17.95 |
| Difference | 1.16 | 1.87 | 0.08 |
| *p* | 0.429 | 0.040 | 0.763 |
|  |  |  |  |
| Actual back transfers from ingroup | 17.85 | 17.69 | 17.95 |
| Actual back transfers from outgroup | 17.09 | 15.81 | 17.87 |
| Difference | 0.76 | 1.87 | 0.08 |
| *p* | 0.047 | 0.040 | 0.763 |
| n | 102 | 39 | 63 |

*Means and differences of investments, expected back transfers and average actual back transfers per group.*

**Table S3** **Group differences in investments, expected and actual back transfers:** Additional tests for statistical significance of differences

|  | All participants | Folk  music fans | Classical music fans |
| --- | --- | --- | --- |
| (1) Investments |  |  |  |
|  |  |  |  |
| Difference in favour of high-status group | + 0.22 | - 2.51 | + 1.90 |
| P-values |  |  |  |
| *Paired t-test* | 0.616 | 0.001 | 0.000 |
| *Wilcoxon matched-pairs signed-ranks test* | 0.315 | 0.001 | 0.000 |
| *Sign test of matched pairs* | 0.350 | 0.002 | 0.000 |
|  |  |  |  |
| Difference in favour of the ingroup | + 2.14 | + 2.51 | + 1.90 |
| P-values |  |  |  |
| *Paired t-test* | 0.000 | 0.001 | 0.000 |
| *Wilcoxon matched-pairs signed-ranks test* | 0.000 | 0.001 | 0.000 |
| *Sign test of matched pairs* | 0.000 | 0.002 | 0.000 |
| (2) Expected back transfers |  |  |  |
|  |  |  |  |
| Difference in favour of high-status group | + 1.33 | - 2.64 | + 3.79 |
| P-values |  |  |  |
| *Paired t-test* | 0.095 | 0.005 | 0.001 |
| *Wilcoxon matched-pairs signed-ranks test* | 0.129 | 0.006 | 0.000 |
| *Sign test of matched pairs* | 0.154 | 0.023 | 0.000 |
|  |  |  |  |
| Difference in favour of the ingroup | + 3.35 | + 2.64 | + 3.79 |
| P-values |  |  |  |
| *Paired t-test* | 0.000 | 0.005 | 0.001 |
| *Wilcoxon matched-pairs signed-ranks test* | 0.000 | 0.006 | 0.000 |
| *Sign test of matched pairs* | 0.000 | 0.023 | 0.000 |
| (3) Actual back transfers |  |  |  |
|  |  |  |  |
| Difference in favour of high-status group | + 1.16 | - 1.87 | + 0.08 |
| P-values |  |  |  |
| *Unpaired t-test^¶^* | 0.429 |  |  |
| *Wilcoxon rank-sum test*^¶^ | 0.155 |  |  |
| *Paired t-test* |  | 0.040 | 0.763 |
| *Wilcoxon matched-pairs signed-ranks test* |  | 0.011 | 0.429 |
| *Sign test of matched pairs* |  | 0.017 | 0.473 |
|  |  |  |  |
| Difference in favour of the ingroup | + 0.76 | + 1.87 | + 0.08 |
| P-values |  |  |  |
| *Paired t-test* | 0.047 | 0.040 | 0.763 |
| *Wilcoxon matched-pairs signed-ranks test* | 0.019 | 0.011 | 0.429 |
| *Sign test of matched pairs* | 0.027 | 0.017 | 0.473 |
| n | 102 | 39 | 63 |

*Differences in investments, expected back transfers and average actual back transfers in favour of the high-status group and in favour of the ingroup. Values are calculated as bivariate mean comparisons and represent how much more (or less) was invested in, expected back from and actually returned from the respective group. Paired t-tests were used to assess the significance of differences. Since differences are not normally distributed, t-tests were complemented with Wilcoxon matched-pairs signed-ranks tests; to account for minor asymmetries in distributions, sign tests of matched pairs were conducted as an additional robustness check. ^¶^The comparison of actual back transfers from folk and classic types over all subjects (not by group) requires tests for unmatched data (t-test for unpaired samples; Wilcoxon rank-sum test) due to the data structure (here: one variable over both types, all other analyses: two variables (one per group)).*

**Table S4 Misjudgements of expected vs. actual back transfers** depending on interaction type (ingroup vs. outgroup encounters)

| Difference expected back transfers – actual back transfers |  |  |  |
| --- | --- | --- | --- |
| *Ingroup encounter* |  | -1.92* |  |
|  |  | (0.75) |  |
| *Investment* |  | 0.46* |  |
|  |  | (0.22) |  |
| *Constant* |  | -7.20*** |  |
|  |  | (1.86) |  |
| n |  | 102 |  |
| N |  | 204 |  |

Standard errors in parentheses; * p<0.05; n = number of clusters = Number of observations

*OLS regression with clustered standard errors. Outcome variable: Divergence between expected back transfers and actual back transfers, computed as the difference between expected back transfers and average actual back transfers for each investment level for both ingroup and outgroup encounters. Independent variables: Dummy for ingroup encounter (= 1; outgroup encounter =0) and investment level (0, 2 …, 18, 20)*

**Table S5** **Investments and expected back transfers *irrespective of counterpart’s identity***

|  | Average  among  folk fans | Average  among  classic fans | Difference |
| --- | --- | --- | --- |
| (1) Investment (averages) | 11.77 | 11.81 | 0.04 |
| *p* |  |  | 0.9712 |
| (2) Expected back transfers (averages) | 17.73 | 17.61 | 0.12 |
| *p* |  |  | 0.9707 |
| n | 39 | 63 | 102 |

*Differences between low- and high-status participants regarding (1) investments irrespectively of the receiver’s identity (averages of all investment decisions) and (2) expected back transfers irrespectively of the sender’s identity (averages of all excepted back transfers). Two sample t-tests were used to assess the significance of differences.*
